# Clinical Features and Outcomes of Very-Early-Onset Inflammatory Bowel Disease in Brazilian Children

**DOI:** 10.1097/PG9.0000000000000032

**Published:** 2020-12-23

**Authors:** Debora Avellaneda Penatti, Nilton Carlos Machado, Mary Assis Carvalho, Maria Aparecida Marchesan Rodrigues

**Affiliations:** From the * Division of Pediatric Gastroenterology and Nutrition, Department of Pediatrics, Botucatu Medical School, Unesp-Sao Paulo State University, Botucatu, São Paulo, Brazil; † Department of Pathology, Botucatu Medical School, Unesp-Sao Paulo State University, Botucatu, São Paulo, Brazil.

**Keywords:** inflammatory bowel disease, ulcerative colitis, Crohn disease, pediatric, very early onset

## Abstract

We report on 20 Brazilian children under 6 years of age with very-early-onset inflammatory bowel disease naive to treatment. The clinical, laboratory, and histopathologic findings at diagnosis and outcomes were reviewed: 13 had ulcerative colitis (UC) and 7 had Crohn disease (CD). The final diagnostic pattern was as follows: 4 children had neonatal-onset (1 UC and 3 CD), 8 had infantile subtype (4 UC and 4 CD), and 8 had UC beyond the neonatal and infantile period. Both forms of inflammatory bowel disease were severe and extensive at diagnosis, with a high prevalence of bloody diarrhea, reflecting the colonic location of the disease. UC was predominantly pancolonic, CD was isolated in the colon and associated with perianal disease. Children with CD were younger than those with UC, were significantly more nutritionally impaired, and had more complications. This study shows that very-early-onset inflammatory bowel disease has an aggressive clinical course with 2 distinct phenotypes, UC and CD, with differences in severity, clinical behavior, and inflammatory pattern but with a preponderance of colonic involvement in both types.

**What Is Known**

- Very-early-onset inflammatory bowel disease (VEO-IBD) is a growing subset of children with inflammatory bowel disease, distinct from that occurring in older children and adults.
- No information is available on the incidence, clinical features, and outcomes of VEO-IBD in developing countries, like Brazil.

**What Is New**

- In Brazilian children, VEO-IBD has an aggressive clinical course with 2 distinct phenotypes, ulcerative colitis and Crohn disease, which differ in severity, clinical behavior, and inflammatory pattern, but both phenotypes have a preponderance of colonic involvement.

**V**ery-early-onset inflammatory bowel disease (VEO-IBD) is a subset of IBD distinct from IBD in older children and adults, with more severe clinical features and more challenging to manage (1–9). The role of monogenic defects has been increasingly recognized in children with VEO-IBD (10). IBD in this younger age group is an increasingly recognized problem, with little information about this pattern of IBD in children from developing countries. Therefore, we analyzed retrospectively the clinical, laboratory, histopathologic findings, and outcomes of 20 Brazilian children with a diagnosis of VEO-IBD. We hypothesized that Brazilian children have different findings at diagnosis of VEO-IBD than those in other countries.

## METHODS

We conducted a retrospective, single-center, descriptive study by chart review of children under 6 years of age, diagnosed with IBD, both ulcerative colitis (UC) and Crohn disease (CD), at the University Hospital Botucatu Medical School (Unesp), São Paulo, Brazil, between January 2000 and February 2020. IBD was diagnosed on the basis of endoscopic and histologic findings by pediatric gastroenterologists and a pathologist (11,12), who provided care following international guidelines (13,14). Clinical data were obtained from electronic medical records using a standard protocol at the initial diagnosis and every 3 months thereafter. VEO-IBD patients were divided according to the Paris classification into 3 age categories: neonate (< 28 d), infantile (age< 2 yr), and very early-onset (<6 yr old). Data were analyzed for statistical significance using GraphPad Prism 7.00 for Windows (GraphPad Software, San Diego, CA). Continuous data are presented as medians with interquartile ranges, and categorical data as absolute numbers and proportions, as appropriate for the distribution normality. Unpaired categorical data were compared using the Fisher exact test. The Mann-Whitney *U* test was used to compare continuous data. All statistical tests were 2-tailed, and a *P* value<0.05 was considered significant. The study was approved by the Institutional Review Board at Botucatu Medical School, Brazil. Written informed consent was obtained from parents or legal caregivers and written assent obtained from children when appropriate.

## RESULTS

Twenty children under 6 years of age were diagnosed with VEO-IBD: 13 children had UC (65%) and 7 had CD (35%); none had a final diagnosis of indeterminate colitis. The diagnostic workup led to the diagnostic pattern of 4 children (1 UC and 3 CD) with neonatal-onset (<28 d), 8 children (4 UC and 4 CD) with infantile subtype (<2 yr), and 8 children with UC beyond the neonatal and infantile period, but less than 6 years of age. The mean follow-up period was 8.3 years (standard deviation = 3.4 yr).

Of the 20 patients diagnosed with VEO-IBD, 17 were female (12 UC and 5 CD) (Table 1). Patients with CD were significantly younger than those with UC (2.5 vs 36 mo; *P* < 0.006). Age at symptoms onset and age at the first visit were significantly lower for patients with CD than for those with UC (Table 1). Four patients (2 UC and 2 CD) had a family history of IBD. One girl with UC had Turner syndrome. Two were dizygotic twins, 1 UC and 1 CD, and their twins were healthy.

**TABLE 1.** Baseline characteristics of children with very-early-onset inflammatory bowel disease

Baseline data	Ulcerative colitis, n = 13	Crohn disease, n = 07	*P*
Sex: female-to-male, n (% female)	12/1 (83)	5/2 (40)	NS*
Age at symptoms onset (mo), median (IQR)	36 (9–55)	2.5 (1–12)	0.006†
Age at first visit, median (IQR)	48 (33–73)	14 (5–28)	0.001†
Symptoms duration at first visit (mo), median (IQR)	12 (3.5–24.5)	5 (4–23)	NS†
IBD family history, n (%)	2 (15)	2 (28)	NS*
Food allergy treatment, n (%)	4 (30)	3 (42)	NS*
Antibiotics for diarrhea, n (%)	9 (69)	7 (100)	NS*
Age of mothers (yr), median (IQR)	37 (32–40)	28 (25–31)	0.02†
Age of fathers (yr), median (IQR)	38 (30–45)	32 (29–39)	NS†
Delivery type: vaginal, n (%)	5 (38)	3 (42)	NS*
Full-term, n (% term)	8 (61)	6 (85)	NS*
Breast feeding, n (%)	13 (100)	7 (100)	NS*
Breast feeding (mo), median (IQR)	6 (4.2–7.5)	4 (1–10)	NS†
Complementary feeding (mo), median (IQR)	6 (6–6)	6 (5–7)	NS†
At birth, median (IQR)			
Weight (g)	3455 (2520–3620)	3000 (2885–3505)	NS†
Height (cm)	49 (48–50)	48 (47–50)	NS†
At first visit, median (IQR)			
Weight *z* score	0.13 (–0.7 to 1.0)	–1.72 (–5.1 to –0.8)	0.003†
Height *z* score	0.09 (–1.4 to 0.9)	–2.44 (–4.2 to –0.6)	0.03†
At 12-mo follow-up, median (IQR)			
Weight *z* score	0.77 (0.6–1.5)	–0.96 (–2.8 to 0.4)	0.03†
Height *z* score	–0.46 (–1.2 to 0.3)	–2.31 (–4.2 to –0.1)	NS†
At 48-mo follow-up, median (IQR)			
Weight *z* score	0.87 (–0.3 to 1.1)	–0.46 (–1.2 to 0.3)	NS†
Height *z* score	–0.58 (–1.5 to 0.1)	–1.69 (–3.0 to –0.3)	NS†

*Fisher exact test.

†Mann-Whitney *U* test.

IBD = inflammatory bowel disease, IQR = interquartile range, NS = not significant.

The proportions of types of delivery and the full-term/premature births were the same for both groups (Table 1). All 20 children were exclusively breastfed for 4 months and were introduced to complementary foods at 6 months. Growth data revealed no significant differences between the 2 groups for weight and height at birth and at 6, 24, 36, and 48 months after diagnosis compared with the reference population. At presentation, the age-adjusted weight and height *z* scores were significantly lower for the patients with CD than for UC. Weight *z* scores remained significantly lower for children with CD at the 12-month follow-up (Table 1). No differences in weight and height *z* scores between the UC and CD groups were detected at the 48-month follow-up.

All children had recurrent episodes of diarrhea, bloody stools, and hospitalization before diagnosis (Table 2). Significant weight loss was observed in patients with CD. Other extraintestinal manifestations, including skin abnormalities, respiratory diseases, and arthritis, were not seen at presentation or during follow-up. One patient with UC developed sclerosing cholangitis during follow-up.

**TABLE 2.** Clinical and laboratory data of children with very-early-onset inflammatory bowel disease

Clinical and laboratory data	Ulcerative colitis, n = 13	Crohn disease, n = 07	*P*
Symptoms and signs, n (%)			
Diarrhea	13 (100)	7 (100)	NS*
Bloody diarrhea	12 (92)	6 (85)	NS*
Abdominal pain	9 (69)	3 (42)	NS*
Weight loss	4 (30)	6 (85)	0.04*
Laboratory			
Hemoglobin, g/dL, median (IQR)	12.2 (10.1–13.0)	9.2 (7.3–9.9)	0.009†
Hemoglobin <11 g/dL, n (%)	04 (30)	07 (100)	0.004*
White blood cell count (10^3^), median (IQR)	9.5 (8.9–15.7)	1.75 (1.0–2.2)	NS†
Eosinophils (n), median (IQR)	270 (85–610)	350 (218–864)	NS†
Platelets (n ×10^3^), median (IQR)	418 (313–565)	482 (277–548)	NS†
CRP, median (IQR)	0.9 (0.5–5.6)	7.4 (3.9–21.9)	0.04†
ESR, mm/h, median (IQR)	23 (12–49)	52 (30–54)	NS†
Total protein, g/dL, median (IQR)	7.4 (6.6–8.2)	6.4 (5.2–6.7)	0.05†
Albumin <3.5 g/dL, n (%)	2 (15)	5 (71)	0.02*
Fecal calprotectin, μg/g, median (IQR)	607 (68–1528)	957 (690–1225)	0.7†
PUCAI/PCDAI moderate to severe at diagnosis, n (%)	12 (92)	07 (100)	NS*

*Fisher exact test.

†Mann-Whitney *U* test.

CRP = C-reactive protein, ESR = erythrocyte sedimentation rate, IQR = interquartile range, NS = not significant, PCDAI = Pediatric Crohn Disease Activity Index, PUCAI = Pediatric Ulcerative Colitis Activity Index.

At presentation, CD patients showed significantly more anemia, higher C-reactive protein (CRP) levels, and low proportion of albumin <3.5 g/dL compared with UC patients (Table 2). White blood cell count, eosinophils, platelets, erythrocyte sedimentation rate, and fecal calprotectin levels did not differ between groups (Table 2). Evaluation of the immune system performed by serum immunoglobulins, lymphocyte subtype counts, granulocyte function, and oxidative burst revealed normal values in both groups. Defects in interleukin-10 signaling and more specific genetic evaluations were not investigated.

Twelve children (92%) with UC and 7 (100%) children with CD had moderate/severe Pediatric Ulcerative Colitis Activity Index (PUCAI) and Pediatric Crohn Disease Activity Index (PCDAI) scores at diagnosis. Both scores significantly decreased after treatment.

The colon was affected in all patients, with pancolitis present in 12 patients with UC and 5 patients with CD. Ileocolonic disease was found in only 1 patient with CD. Perianal lesions were present in 6 patients with CD and ranged from single simple skin tags to complex networks of fistulas and abscesses. Perianal fistulae were detected in 5 patients with CD. According to the Paris classification, 12 children with UC were classified as E3 (pancolitis), and 6 children with CD were classified as L2 (colon only).

Histologic findings of the 2 groups were significantly different. Diffuse inflammation with cryptitis was observed in all 13 children with UC and in only 1 child with CD. Crypt microabscesses were detected in 10 patients with UC and in 1 patient with CD. Crypt distortion was present in 11 patients with UC and in 1 patient with CD. Plasmacytosis was present in 12 patients with UC and in 1 patient with CD. Epithelioid microgranulomas were detected in 3 children with CD but in no patient with UC.

Patients in both groups (13 UC and 7 CD) were initially treated with mesalamine and oral corticosteroids but were refractory to the conventional therapy. Remission was induced with systemic corticosteroids in all children. A high proportion of both groups (85% of children with CD and 76% of children with UC) started immunosuppressant therapy with azathioprine (2 mg/kg/d). Infliximab (5 mg/kg) was administered to 42% of the children with CD and 15% of those with UC and they did well with these treatment options.

Two children with CD developed non-Hodgkin lymphoma; both were diffuse large B-cell lymphomas. These CD children were on concomitant treatment with azathioprine and infliximab due to isolated colonic disease and severe perianal lesions. One child was considered cured at the 3-year follow-up and the other child underwent a total colectomy during treatment for lymphoma and died in the postoperative period. Another patient with neonatal-onset CD who had severe perianal lesions died of septicemia. Monogenic defects were not investigated in these children. No other surgical procedures were required for any of the 18 surviving children during follow-up.

## DISCUSSION

This study was a retrospective single-center analysis of clinical, laboratory, histologic findings, and outcomes of 20 Brazilian children under 6 years of age at the first diagnosis of VEO-IBD. The small number of cases encountered over 2 decades corresponds to 15% of all IBD pediatric cases diagnosed in our service. It is noteworthy that our center is the only in the state region that provides tertiary care for children with IBD.

The predominance of UC over CD in this age group has been reported previously (3,5,9). No patient had the final diagnosis of indeterminate colitis, probably because the long interval between first symptoms and diagnosis precluded misclassification upon evaluation (7). One patient was initially diagnosed with UC, due to the presence of pancolitis, with all morphological features of UC. On follow-up, he developed perianal disease, which led to the correct diagnosis of CD.

Both forms of IBD were severe and extensive at diagnosis, with a high prevalence of bloody diarrhea that reflected the colonic location of the disease, similar to findings in previous reports (6,9,15). However, in our study, significant differences were noted between those with UC and with CD. The patients with CD were younger than those with UC, with the majority (57%) under 2 years of age. At the initial visit, children with CD presented with weight loss indicating significant nutritional impairment. This agrees with the study by Mamula et al (6), which found weight loss and failure to grow in children with CD compared with children with UC. The children in our study with CD had hypoalbuminemia. A lower albumin level may be a proxy for more severe disease in younger children in terms of nutrition. This agrees with the *z* score for the lower pooled weight for age observed in our younger group of children with CD. Oral and enteral nutritional therapy was necessary for 85% of the children with CD. These findings are similar to those of previous reports (9,16).

Perianal disease was reported in 6 of 7 children (85%) with CD and in none with UC, confirming the importance of perianal disease in the diagnosis of CD. We did not find extraintestinal manifestations of IBD in children with CD, in contrast to Ledder et al (9), who reported extraintestinal manifestations in 75% of the children in their study with VEO-IBD. The clinical severity of VEO-IBD at first diagnosis, as indicated by the PUCAI and PCDAI scores, was classified as moderate/severe in all 20 children. Both scores significantly decreased after treatment and were indispensable in evaluating disease activity, especially the PUCAI. Abdominal pain was reported in children from both groups, similar to those reported by Mamula et al (6) but in higher proportion than those reported by Ledder et al (9). However, a history of abdominal pain is often unreliable for children in this age group (2).

Our review found more anemia in the patients with CD than previously reported (9,16). Thrombocytosis occurred in a greater proportion of the children with CD, similar to that found in previous studies (9,16). CRP levels helped evaluate inflammatory activity because all children with CD had increased CRP levels at the first visit. Kammermeier et al (15) found a similar increase in CRP levels of children with VEO-IBD. The erythrocyte sedimentation rate data were similar. The median fecal calprotectin level was higher in children with CD than in UC (957 vs 607 μg/g), but the difference was not significant. Therefore, reduced hemoglobin levels, elevated platelet count, increased fecal calprotectin markers, and reduced serum albumin were suggestive of IBD at the time of diagnosis (Table 2).

Our study had only 1 patient with CD with ileocolonic involvement, similar to previous studies (2–4,17). Van Limbergen et al (8) found that children with CD and under 8 years of age at the initial diagnosis were more likely than older children with CD to have isolated colonic disease and less likely to have small bowel involvement. They also found that younger children with UC had more extensive colitis than did older children. Our study confirmed the high prevalence of isolated colonic disease in patients with UC and CD. According to the Paris classification, 12 children with UC were classified as E3 (pancolitis) and 6 children with CD were classified as L2 (colon only). The proportion of pancolitis in patients with UC and CD was similar to that reported by Ledder et al (9) and reinforces that VEO-IBD has a more severe phenotype in this age group than in older children, adolescents, and adults, as found in other studies (8,9).

Because CD in patients under 6 years of age is located mainly in the colon, it is difficult to distinguish UC from CD. In our study, histopathologic evaluation was useful in discriminating between UC and CD. Focal active inflammation, absence of crypt distortion, and epithelioid microgranulomas are features of CD (11). Diffuse inflammation, architectural distortion, cryptitis, crypt microabscesses, and plasmacytosis are characteristic of UC (12). In most of patients with UC, we found chronic changes such as architectural distortion and plasmacytosis, in contrast to the findings of Robert et al (18), who observed few histologic abnormalities in children with UC at initial presentation. This difference may be related to the long interval between initial symptoms and diagnosis of IBD in our patients.

Lymphoma occurred in 2 children with CD on concomitant azathioprine and infliximab therapy. Both were non-Hodgkin diffuse large B-cell lymphomas and may be related to the risk of lymphoproliferative disease in children with IBD who are treated with a combination of thiopurines and anti-tumor necrosis factor drugs. Monogenic defects were not evaluated in these children, and this is one of the main limitations of our study.

In conclusion, VEO-IBD in a population of children in Brazil is similar to that reported in other countries, both have an aggressive clinical course with 2 distinct phenotypes, UC and CD, which differ in severity, clinical behavior, and inflammatory pattern, but have a preponderance of colonic involvement.
